# Efficacy and Safety of Anticoagulation Treatment in COVID-19 Patient Subgroups Identified by Clinical-Based Stratification and Unsupervised Machine Learning: A Matched Cohort Study

**DOI:** 10.3389/fmed.2021.786414

**Published:** 2021-12-24

**Authors:** Yi Bian, Yue Le, Han Du, Junfang Chen, Ping Zhang, Zhigang He, Ye Wang, Shanshan Yu, Yu Fang, Gang Yu, Jianmin Ling, Yikuan Feng, Sheng Wei, Jiao Huang, Liuniu Xiao, Yingfang Zheng, Zhen Yu, Shusheng Li

**Affiliations:** ^1^Department of Emergency Medicine, Tongji Medical College, Tongji Hospital, Huazhong University of Science and Technology, Wuhan, China; ^2^Department of Critical Care Medicine, Tongji Medical College, Tongji Hospital, Huazhong University of Science and Technology, Wuhan, China; ^3^Germany Research Center for Artificial Intelligence, Saarland Informatics Campus, Saarbrücken, Germany; ^4^Intelligent Medicine Research Center, Greater Bay Area Institute of Precision Medicine (Guangzhou), Fudan University, Guangzhou, China; ^5^Department of Neurology, Tongji Medical College, Tongji Hospital, Huazhong University of Science and Technology, Wuhan, China; ^6^Ministry of Education Key Laboratory of Environment and Health, Department of Epidemiology and Biostatistics, School of Public Health, Tongji Medical College, Huazhong University of Science and Technology, Wuhan, China; ^7^Center for Evidence-Based and Translational Medicine, Zhongnan Hospital of Wuhan, Wuhan, China; ^8^Department of Critical Care Medicine, Renmin Hospital of Wuhan University, Wuhan, China

**Keywords:** COVID-19, anticoagulation, outcomes, mortality, bleeding events, unsupervised machine learning

## Abstract

**Objective:** To explore the efficacy of anticoagulation in improving outcomes and safety of Coronavirus disease 2019 (COVID-19) patients in subgroups identified by clinical-based stratification and unsupervised machine learning.

**Methods:** This single-center retrospective cohort study unselectively reviewed 2,272 patients with COVID-19 admitted to the Tongji Hospital between Jan 25 and Mar 23, 2020. The association between AC treatment and outcomes was investigated in the propensity score (PS) matched cohort and the full cohort by inverse probability of treatment weighting (IPTW) analysis. Subgroup analysis, identified by clinical-based stratification or unsupervised machine learning, was used to identify sub-phenotypes with meaningful clinical features and the target patients benefiting most from AC.

**Results:** AC treatment was associated with lower in-hospital death risk either in the PS matched cohort or by IPTW analysis in the full cohort. A higher incidence of clinically relevant non-major bleeding (CRNMB) was observed in the AC group, but not major bleeding. Clinical subgroup analysis showed that, at admission, severe cases of COVID-19 clinical classification, mild acute respiratory distress syndrome (ARDS) cases, and patients with a D-dimer level ≥0.5 μg/mL, may benefit from AC. During the hospital stay, critical cases and severe ARDS cases may benefit from AC. Unsupervised machine learning analysis established a four-class clustering model. Clusters 1 and 2 were non-critical cases and might not benefit from AC, while clusters 3 and 4 were critical patients. Patients in cluster 3 might benefit from AC with no increase in bleeding events. While patients in cluster 4, who were characterized by multiple organ dysfunction (neurologic, circulation, coagulation, kidney and liver dysfunction) and elevated inflammation biomarkers, did not benefit from AC.

**Conclusions:** AC treatment was associated with lower in-hospital death risk, especially in critically ill COVID-19 patients. Unsupervised learning analysis revealed that the most critically ill patients with multiple organ dysfunction and excessive inflammation might not benefit from AC. More attention should be paid to bleeding events (especially CRNMB) when using AC.

## Introduction

Coronavirus disease 2019 (COVID-19), caused by severe acute respiratory syndrome coronavirus 2 (SARS-CoV-2), has developed into a pandemic disease and affected nearly every country in the world. There is no comprehensive and strong clinical evidence to support the efficacy of any drugs that specifically target the SARS-CoV-2 (1). Previous research has found that coagulopathy is very common in COVID-19 patients, and includes thrombosis and coagulation abnormalities and dysfunction such as an elevated D-dimer level and prolonged prothrombin time (PT), respectively (2). Autopsy histopathologic analysis has identified widespread thrombosis and microangiopathy in small vessels and capillaries of the lung (3–5), which are different from the pathologies observed in respiratory failure caused by other diseases (3, 6–8). Some scholars have therefore proposed anticoagulation (AC) treatment as an integral part of systemic therapy in the early stage of COVID-19 (9). Generally, retrospective studies have suggested that AC may decrease mortality in COVID-19 patients (9, 10), However, these conclusions are not completely reliable nor applicable to all COVID-19 patients due to limitations in methodology such as no prospective control or matching cohort (9–11), large heterogeneity in anticoagulant therapy (9–12), and a lack of subgroup analysis (10, 11, 13). A recently completed randomized controlled trial (RCT) found that, compared with usual-care thromboprophylaxis, an initial strategy of therapeutic-dose anticoagulation did not result in a higher probability of survival in critically ill COVID-19 patients (14). The conclusion of this RCT may be inconsistent with that of previous retrospective studies. At present, the recommendations for empiric systemic AC treatment currently differ between COVID-19 management guidelines (15–17), with some recommending using anticoagulant drugs preventively for patients who have no contraindications to AC and a significantly increased D-dimer level, while others recommend that all hospitalized adults with COVID-19 should receive pharmacologic thromboprophylaxis with low molecular weight heparin (LMWH) rather than unfractionated heparin (UFH).

We conducted a retrospective cohort study using a comprehensive database of COVID-19 patients to investigate whether AC treatment was protective and safe for COVID-19 patients. Innovative analyses using propensity score matching (PSM) and inverse probability of treatment weighting (IPTW) were performed to balance baseline covariates, variates related to AC treatment assignment and variates related to the outcome between patients with or without AC treatment. Further sensitivity analyses were carried out to explore the association between outcome and duration, dosage and type of AC treatment. The second aim of the study was to identify the patients who benefited most from AC treatment using subgroup analysis that involved stratifying the data according to the severity of the acute respiratory distress syndrome (ARDS) (18), COVID-19 clinical classification (17), and D-dimer levels. Taking into account the heterogeneity of the patients, clinically relevant patient subpopulations were identified by unsupervised machine learning algorithms. The effectiveness of AC treatment was verified further in identified clusters.

## Materials and Methods

### Ethics and Registration

This retrospective cohort study was approved by the ethics committee of Tongji Medical College, Huazhong University of Science and Technology (No. 2020-S220). The clinical trial was registered and verified by the Chinese Clinical Trial Registry (ChiCTR2000039855).

### Patient Population

This single-center retrospective cohort study was conducted in two designated branches for COVID-19 patients in Tongji Hospital, an academic hospital affiliated to Tongji Medical College, Huazhong University of Science and Technology in Wuhan, China. All patients with confirmed COVID-19 admitted consecutively to these two institutions between Jan 25 to Mar 23, 2020, were enrolled retrospectively in the study. Approval was obtained from the ethics committee at our institution that the patients did not need to provide informed consent for inclusion in the study. Patients were assigned to three groups, including one group of patients with systemic AC treatment for at least 7 days, one group of patients with systemic AC treatment for <7 days and one group of patients without AC treatment. The medications administered and clinical outcomes were followed up to June 4, 2020, when these two branches for exclusive COVID-19 treatment were closed. All COVID-19 patients were diagnosed according to the World Health Organization interim guidelines (19) and the Diagnosis and Treatment Protocol for COVID-19 Patients (Trial Version 8) (17). The exclusion criteria for the study were younger than 18 years, pregnant, length of stay <24 h, insufficient medical information, a history of severe comorbidities requiring surgical operation including, but not limited to, multiple trauma, a severe infection that required debridement, amputation or laparotomy, and patients who were classified again as COVID positive after RNA for SARS-CoV-2 was detected following their discharge from hospital.

### Anticoagulation Exposure

AC treatment was defined as receiving either UFH, LMWH, Fondaparinux sodium, Argatroban, or direct-acting oral anticoagulants (DOACs) (mainly Rivaroxaban). The initiation of AC treatment was decided by the bedside physicians. Possible reasons for AC treatment were extracted from electronic case files. Immortal time is a gap period between exposure and initiation of follow-up (20). We carried out a Cox proportional hazards model with a time-dependent manner for the drug exposure in this study.

### Outcomes, Definitions, and Data Collection

The primary outcome of this study was in-hospital mortality. The safety endpoints included bleeding events and thrombocytopenia. Major bleeding was defined according to the International Society on Thrombosis and Haemostasis (ISTH) statement (21) as those that resulted in death, were life-threatening, caused chronic sequelae, or consumed major healthcare resources. Hemorrhage that did not fit the criteria for the ISTH definition of major bleeding but required medical intervention was classified as clinically relevant non-major bleeding (CRNMB) (22). Other bleeding events which did not meet the criteria of either major bleeding or CRNMB, including bloody sputum, positive fecal occult blood test/gastric occult blood test and microscopic hematuria, were reported separately. Thrombocytopenia was defined as a platelet count <100 × 10^9^/L (23).

The CURB-65 score (21, 24), ARDS (18, 25, 26), and quick sequential organ failure assessment (qSOFA) (27) were defined according to the literature, while the COVID-19 clinical classification was made according to the Diagnosis and Treatment Protocol for COVID-19 Patients (Trial Version 8) (17). The detailed definition of ARDS and COVID-19 clinical classification is shown in Supplementary Table 1.

All the characteristics and clinical information of the patients were obtained from electronic medical and nursing record systems. This data included age, gender, current smoking history, comorbidities, laboratory results at admission, CURB-65 score and qSOFA score at admission, ARDS classification (18) and COVID-19 clinical classification at admission and during the hospital stay, antiviral therapies and other treatments during hospitalization, the level of oxygen therapy at admission, and the most intense level of oxygen therapy during hospitalization. Variables with missing data >20% were excluded from this analysis. Multiple imputations were conducted to address the presence of missing values.

### Unsupervised Clustering

For this work, we used the K-Medoids clustering algorithm to partition our data into subclasses in an unsupervised manner. The K-Medoids algorithm randomly selects K samples in the training data as the medoids. The remaining samples are assigned to each subclass based on the pairwise dissimilarities. The sample, which is more similar to the medoid, is assigned to the corresponding subclass. Next, the medoids are updated based on the new results of subgrouping. These two steps are iterated multiple times until there is no change in the assignments. In particular, we used the Partitioning Around Medoids (PAM), which is the most common implementation of K-Medoids. For the dissimilarity measure, we adopted the Manhattan distance because it has better performance than the Euclidean distance for the data containing both binary and category variables (28).

A total of 25 variables representing the patients' clinical characteristics were used as input features in the unsupervised learning method, which included demographic features, comorbidities, vital signs, biomarkers, and oxygen therapy types at admission and during the hospital stay. Each patient in our database was presented as a vector with 25 dimensions. To prepare each patient's case as a vector for modeling training, we converted the binary variable as (0, 1), with the category variable represented by the corresponding categorical index. Normalization of the numerical variables was performed. After the normalization, we made sure that each numerical variable had a normal distribution.

Two prominent probabilistic model selection methods: Bayesian information criterion (BIC) and Akaike information criterion (AIC) were used to determine the optimal number of clusters in this work. In general, the measurement of AIC and BIC scores are similar. BIC penalizes the complexity of the model more than AIC (29).

### Statistical Analysis

To minimize bias caused by the non-random allocation of potentially confounding covariates between the AC and non-AC groups, we adopted PSM methods (30). Propensity score (PS) was calculated using a logistic regression model, adjusted for the following covariates: level of oxygen therapy, clinical classification, high-sensitivity C reactive protein (hs-CRP), D-dimer levels, platelets count, CURB-65 score for the severity of pneumonia (31) at hospital admission, and the highest level of oxygen therapy during hospitalization. The match ratio was set at 1 to 3 and the maximum allowable distance (caliper) at 0.1 (32). To detect the selective bias potentially caused by this PSM, inverse probability of treatment weighting analysis (IPTW) was carried out based on the same variates used in PSM modeling (33).

Continuous variables were expressed as medians and interquartile ranges (IQR) and compared using the Mann-Whitney U test or Kruskal-Wallis H test. Categorical variables were compared using the Pearson χ2 test, continuity correction, or Fisher's exact test, as appropriate. Differences between clusters identified by unsupervised machine learning were judged by two-tailed Bonferroni correction *post hoc* tests, with a *P*-value < (0.05/6) considered statistically significant. A Kaplan-Meier curve was used to analyze survival during hospitalization, with the data stratified according to AC treatment and PAM clustering subphenotypes.

Univariate and multivariate Cox proportional hazards regression was used to determine the risk factors for in-hospital mortality in the PS matched cohort. Residual imbalanced variates were included in the multivariate Cox proportional hazards regression for the PSM cohort. Cox regression analysis with IPTW adjusted covariates of important demographic characteristics as well as variates associated with outcomes either reported previously (34–36) or by general clinic consensus, which included age, gender, platelets count, PT, D-dimer, total bilirubin, lactate dehydrogenase, urea and hs-CRP. Competing risk model analyses were carried out using Fine-Gray tests, which considered death as a competing event for the safety endpoints, including bleeding events and thrombocytopenia. Sensitivity analyses were carried out according to the AC exposure duration, type and dosage in the full cohort. In subgroups analysis among PS matched cohort, univariate and multivariate logistic regression analysis was used to explore the association of outcomes and AC treatment. Residual imbalanced variates were included in the multivariate logistic regression here. The interaction effect between AC treatment and subgroups was also analyzed by logistic regression.

SPSS version 26.0 software (IBM Corp., Armonk, New York, USA) and SAS version 9.4 (SAS Institute Inc. Cary, NC, USA) were used for the statistical analyses and PS matching. The Kaplan-Meier survival plot and forest plot were constructed using GraphPad Prism version 4.0 software (GraphPad Software Inc., La Jolla, CA, USA). All tests were two-tailed, with a *P* < 0.05 considered statistically significant.

## Results

### Clinical Characteristics of the Patients at Presentation

**Figure 1:**
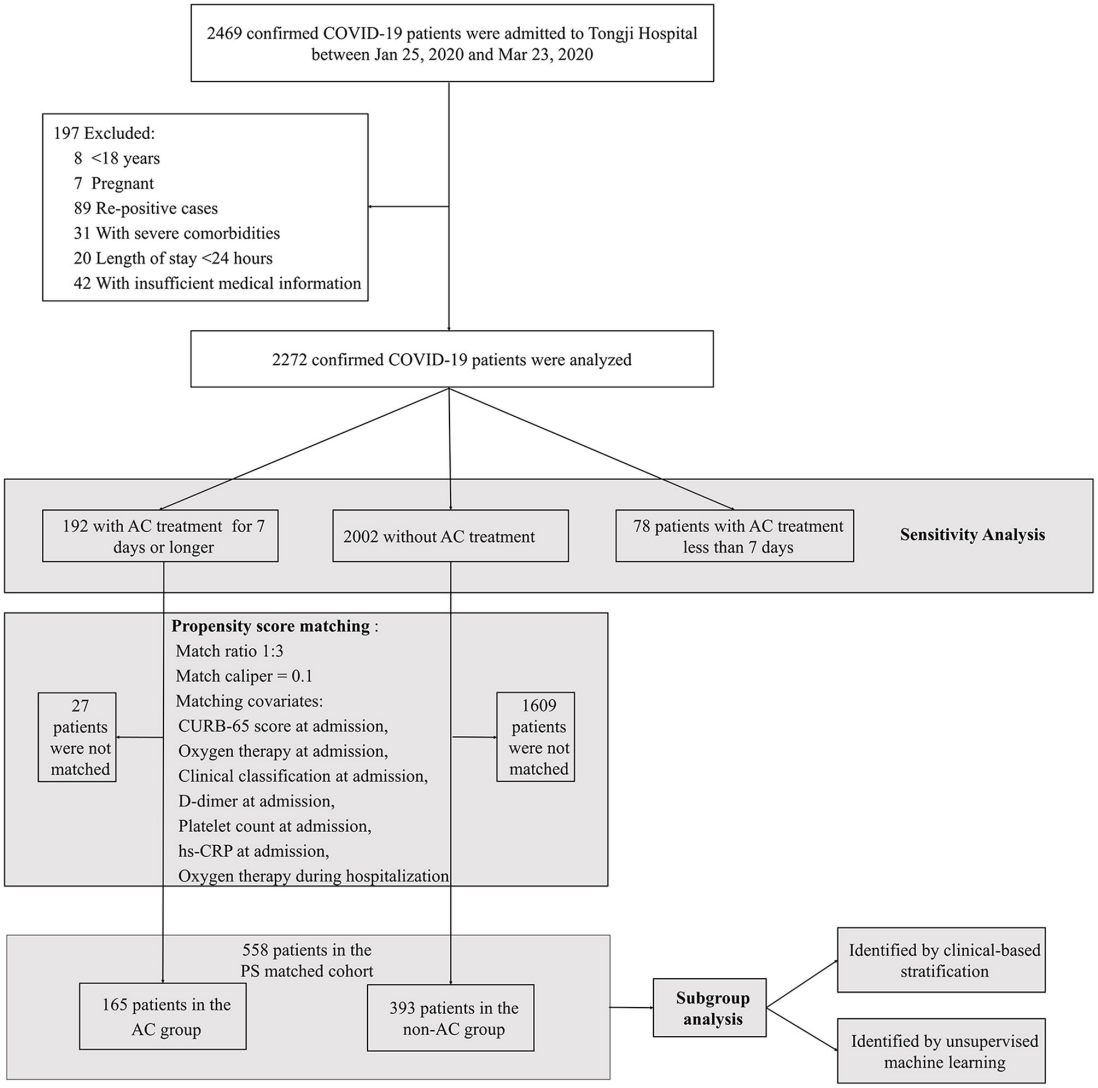
Flow diagram of the study. COVID-19, Coronavirus Disease 2019; AC, anticoagulation; hs-CRP, high sensitivity C-reactive protein; PS, propensity score.

Two thousand four hundred and sixty nine confirmed COVID-19 patients admitted to Tongji Hospital between Jan 25 and Mar 23, 2020, were consecutively and unselectively reviewed. After excluding 197 patients who did meet study exclusion criteria, a total of 2,272 patients were identified for IPTW analysis and sensitivity analyses. In PSM modeling, 78 patients who received AC treatment for <7 days were not included for matching. Finally, PS matching yielded 165 patients in the AC group (patients who received AC for 7 days or longer) and 393 in the non-AC group (patients who did not receive AC) (Figure 1). Detailed AC treatment type, dosage, duration, time of initiation from admission and possible reasons for AC treatment were shown in Supplementary Figure 1. In the PS matched cohort, compared to the non-AC group, patients in the AC group were older (69 years, interquartile range [IQR] 60–78 vs. 65 years, IQR 53–71 years, *P* < 0.001) and had more comorbidities at admission (75.2% vs. 58.5%, *P* < 0.001, Table 1).

**Table 1:** Patients Baseline Characteristics and Treatments in propensity score matched cohort.

	**No. (%)**			
	**Total (*n* = 558)**	**AC treatment (*n* = 165)**	**Non-AC treatment (*n* = 393)**	***P*** **-value**
Age, median (IQR), years	66 (56, 73)	69 (60, 78)	65 (53, 71)	<0.001
**Gender**				
Male	307 (55.0)	91 (55.2)	216 (55.0)	0.967
Female	251 (45.0)	74 (44.8)	177 (450)	
Current smoking	40 (7.2)	18 (10.9)	22 (5.6)	0.026
Comorbidities	354 (63.4)	124 (75.2)	230 (58.5)	<0.001
Diabetes	118 (21.1)	41 (24.8)	77 (19.6)	0.165
Hypertension	247 (44.3)	90 (54.5)	157 (39.9)	0.002
Cardiovascular disease	71 (12.7)	31 (18.8)	40 (10.2)	0.005
CODP	45 (8.1)	20 (12.1)	25 (6.4)	0.023
Chronic kidney disease	19 (3.4)	6 (3.6)	13 (3.3)	0.845
Chronic liver disease	18 (3.2)	4 (2.4)	14 (3.6)	0.487
Autoimmune disease	8 (1.4)	2 (1.2)	6 (1.5)	1.000
Immunosuppression	2 (0.4)	0 (0)	2 (0.5)	1.000
Malignancy	21 (3.8)	3 (1.8)	18 (4.6)	0.118
**Oxygen therapy at admission**				
Without oxygen inhalation	100 (17.9)	25 (15.2)	75 (19.1)	0.206
Nasal cannula	375 (67.2)	110 (66.7)	265 (67.4)	
Face mask with reservivor bag	46 (8.2)	20 (12.1)	26 (6.6)	
High-flow nasal cannula	4 (0.7)	2 (1.2)	2 (0.5)	
Non-invasive ventilation(bi-level)	20 (3.6)	4 (2.4)	16 (4.1)	
Invasive mechanical ventilation	13 (2.3)	4 (2.4)	9 (2.3)	
**ARDS at admission**				
No ARDS	331 (59.3)	85 (51.5)	246 (62.6)	0.067
Mild	120 (21.5)	45 (27.3)	75 (19.1)	
Moderate	71 (12.7)	25 (15.2)	46 (11.7)	
Severe	36 (6.5)	10 (6.1)	26 (6.6)	
**Clinical classification at admission**				
Moderate	62 (11.1)	18 (10.9)	44 (11.2)	0.537
Severe	459 (82.3)	139 (84.2)	320 (81.4)	
Critical	37 (6.6)	8 (4.8)	29 (7.4)	
CURB-65 score at admission	1 (0, 2)	1 (0, 2)	1 (0, 1)	0.099
qSOFA score at admission	0 (0, 1)	0 (0, 1)	0 (0, 1)	0.608
**Initial laboratory parameters, median (IQR)**				
White blood cells, × 10^9^/L	6.29 (4.89, 8.58)	7.15 (5.40, 9.57)	5.97 (4.60, 8.02)	<0.001
Neutrophils, × 10^9^/L	4.71 (3.12, 7.04)	5.65 (4.06, 7.78)	4.24 (2.90,6.37)	<0.001
Lymphocytes, × 10^9^/L	0.90 (0.60, 1.31)	0.84 (0.60, 1.15)	0.94 (0.61, 1.34)	0.060
Platelets, × 10^9^/L	217.0 (153.0, 291.3)	209.0 (143.5, 278.0)	219.0 (159.0, 293.0)	0.129
Total bilirubin, mmol/L	9.6 (7.2, 13.7)	10.7 (7.0, 14.8)	9.4 (7.2, 13.1)	0.072
Lactate dehydrogenase, U/L	321.0 (237.0, 450.3)	357.0 (262.5, 468.0)	313.0 (228.0, 434.5)	0.002
Urea, mmol/L	5.0 (3.6, 7.0)	5.2 (4.1, 7.4)	4.8 (3.4, 6.8)	0.019
hs-CRP, mg/L	43.6 (13.0, 102.0)	50.9 (1.0, 112.7)	36.9 (10.6, 99.0)	0.009
Prothrombin time, s	14.0 (13.4, 15.0)	14.1 (13.5, 15.2)	14.0 (13.4, 14.9)	0.142
D-dimer, mg/mL	1.36 (0.58, 2.98)	2.22 (1.11, 6.21)	1.11 (0.56, 2.58)	<0.001
Creatine	72 (58, 89)	74 (59, 90)	71 (58, 89)	0.707
Antiviral therapy	522 (93.5)	162 (98.2)	360 (91.6)	0.004
**Other treatments**				
Intravenous immunoglobulin	186 (33.3)	82 (49.7)	104 (26.5)	<0.001
Corticosteroid	276 (49.5)	107 (64.8)	169 (43.0)	<0.001
Convalescent plasma	22 (3.9)	15 (9.1)	7 (1.8)	<0.001
**Oxygen therapy in hospitalization**				
Without oxygen inhalation	6 (1.1)	1 (0.6)	5 (1.3)	<0.001
Nasal cannula	328 (58.8)	85 (51.5)	243 (61.8)	
Face mask with reservivor bag	43 (7.7)	15 (9.1)	28 (7.1)	
High-flow nasal cannula	25 (4.5)	8 (4.8)	17 (4.3)	
Non-invasive ventilation(bi-level)	79 (14.2)	15 (9.1)	64 (16.3)	
Invasive mechanical ventilation	72 (12.9)	36 (21.8)	36 (9.2)	
ECMO	5 (0.9)	5 (3.0)	0 (0)	

*Variables represented the poorest value of the first day at admission. AC, anticoagulation; IQR, interquartile range; COPD, Chronic obstructive pulmonary disease; ARDS, acute respiratory distress syndrome; qSOFA, quick sequential organ failure assessment; hs-CRP, high sensitive C reacting protein; ECMO, extracorporeal membrane oxygenation*.

### Primary and Secondary Outcomes

**Figure 2:**
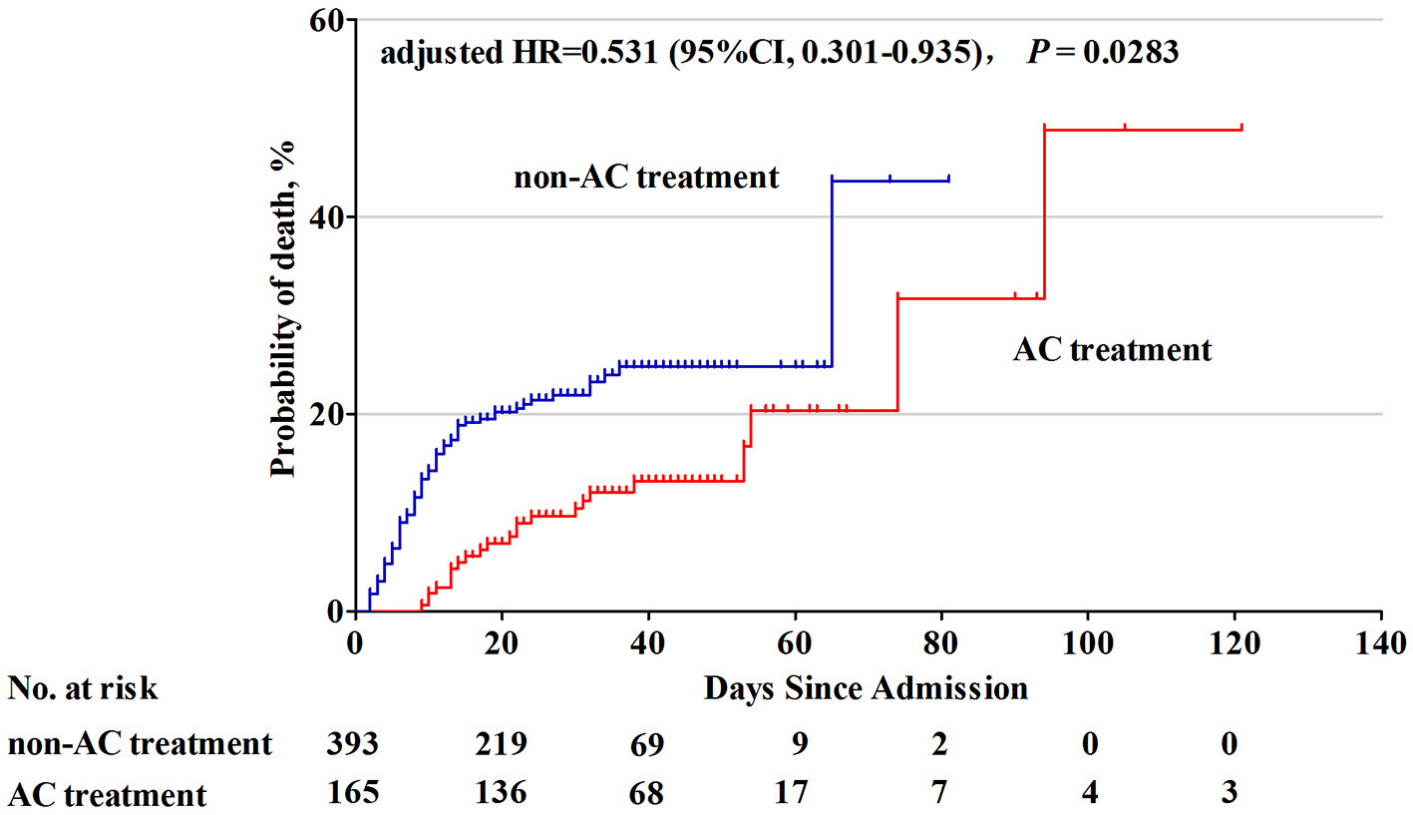
Cumulative probability of death in COVID-19 patients with and without AC treatment. Patients with AC treatment had a lower probability of in-hospital mortality than those without AC treatment (adjusted* HR = 0.531, 95% CI: 0.301–0.935, *P* = 0.0283). *Adjusted for time-dependent AC exposure and baseline covariates including age, smoking, comorbidities, white blood cells, D-dimer, lactate dehydrogenase, urea, antiviral therapy, intravenous immunoglobulin, convalescent plasma therapy, and oxygen therapy in hospitalization. HR, hazard ratio; CI, confidence interval; AC, anticoagulation.

In the PS matched cohort, univariate Cox proportional hazard regression analysis showed a significantly lower probability of in-hospital death in the AC treatment group compared to that in the non-AC treatment group (hazard ratio [HR] = 0.450; 95% confidence interval [CI], 0.278 to 0.727; *P* < 0.001). Since there were still residual imbalances between AC and non-AC groups, multivariate Cox proportional hazard regression was carried out by adjusting imbalance covariates, including age, smoking, comorbidities, white blood cells, lactate dehydrogenase, urea, D-dimer, antiviral therapy, intravenous immunoglobulin, and oxygen therapy during hospitalization. Multivariate Cox analysis showed that AC treatment was associated with a lower probability of in-hospital mortality (adjusted HR = 0.249, 95% CI 0.143 to 0.436, *P* < 0.001, Table 2). To explore the immortal time bias, a further Cox proportional hazards model with a time-dependent manner for AC exposure was carried out in this study. This showed that the AC treatment was still associated with a lower probability of in-hospital death risk in the PS matched cohort (adjusted HR = 0.531, 95% CI 0.301 to 0.935, *P* = 0.028, Figure 2).

**Table 2:** Primary and secondary outcomes of PS matched cohort.

	**No. (%)**					
	**Total** **(*n* = 558)**	**AC group** **(*n* = 165)**	**Non-AC group** **(*n* = 393)**	**Crude HR** **(95% CI)**	Adjusted^*^ HR **(95% CI)**	Adjusted^*^ HR for time-dependent AC exposure **(95% CI)**
**Primary outcomes**						
In-hospital mortality	107 (19.2)	23 (13.9)	84 (21.4)	0.450 (0.278–0.727)	0.249 (0.143–0.436)	0.531 (0.301–0.935)
**Secondary outcomes**						
Bleeding events	121 (21.7)	42 (25.5)	79 (20.1)	0.673 (0.460–0.984)	0.675 (0.413–1.104)	3.187 (1.846–5.504)
Major bleeding	4 (0.7)	0 (0)	4 (1.0)	0.017 (0–58.149)	–	–
CRNMB	32 (5.7)	17 (10.3)	15 (3.8)	1.339 (0.663–2.705)	1.149 (0.465–2.839)	3.713 (1.446–9.532)
Bloody sputum	9 (1.6)	3 (1.8)	6 (1.5)	0.628 (0.155–2.544)	0.201 (0.008–4.896)	0.958 (0.051–18.063)
Positive FOBT/GOBT	5 (0.9)	4 (2.4)	1 (0.3)	4.011 (0.447–35.976)	–	–
Microscopic hematuria	80 (14.3)	25 (15.2)	55 (14.0)	0.597 (0.370–0.965)	0.443 (0.232–0.846)	2.624 (1.280–5.380)
Thrombocytopenia	79 (14.2)	25 (15.2)	54 (13.8)	0.608 (0.372–0.993)	0.665 (0.334–1.327)	2.167 (0.750–6.263)

* *Adjusted for baseline covariates including age, smoking, comorbidities, white blood cells, D-dimer, lactate dehydrogenase, urea, antiviral therapy, intravenous immunoglobulin, convalescent plasma therapy, and oxygen therapy in hospitalization. PS, propensity score; AC, anticoagulation; HR, hazard ratio; CI, confidence interval; CRNMB, clinically relevant non-major bleeding; FOBT, fecal occult blood test; GOBT, gastric occult blood test*.

To detect the selective bias caused by PSM, IPTW analysis was performed in the full cohort including 2,272 patients. Multivariate Cox analysis again showed that AC treatment was associated with a lower probability of in-hospital mortality by comparing patients without AC treatment and patients with AC treatment for 7 days or longer (adjusted HR = 0.164; 95% CI: 0.104 to 0.260, *P* < 0.001). Sensitivity analyses were carried out according to the AC exposure duration, type and dosage in the full cohort. Duration of AC therapy <7 days was not associated with lower in-hospital mortality (adjusted HR = 1.018; 95% CI: 0.742 to 1.399, *P* = 0.910). In addition, AC treatment in all various dosages and types remained consistently associated with lower mortality (Table 3).

**Table 3:** Hazard ratio for in-hospital mortality in the full cohort by AC treatment duration, dosage and type in sensitivity analyses.

	**No. of in-hospital death/total no. (%)**	**Crude HR (95% CI)**	Adjusted^*^ HR (95% CI)	Adjusted^*^ HR for IPTW model^#^ (95% CI)
Non-AC treatment	91/2002 (4.5)	Reference	Reference	Reference
**Duration**				
AC treatment <7 days	44/78 (56.4)	13.600 (9.482–19.507)	3.424 (2.242–5.230)	1.018 (0.742–1.399)
AC treatment for 7 days or longer	38/192 (19.8)	2.881 (1.943–4.272)	0.864 (0.560–1.335)	0.164 (0.104–0.260)
**Dosage**				
Low dose thromboprophylaxis	43/176 (24.4)	4.354 (3.020–6.277)	1.387 (0.923–2.085)	0.498 (0.329–0.754)
Intermediate dose thromboprophylaxis	21/55 (38.2)	6.702 (4.153–10.816)	1.492 (0.870–2.560)	0.349 (0.224–0.545)
Therapeutic dose anticoagulation	18/39 (46.2)	6.959 (4.131–11.723)	1.575 (0.905–2.742)	0.225 (0.132–0.384)
**Type**				
LMWH	80/262 (30.5)	5.120 (3.878–7.162)	1.432 (1.004–2.043)	0.382 (0.271–0.537)
Non-LMWH	2/8 (25.0)	3.590 (0.866–14.884)	2.029 (0.482–8.538)	0.094 (0.019–0.466)

* *Adjusted for baseline covariates including age, gender, levels of platelets count, prothrombin time, D-dimer, total bilirubin, lactate dehydrogenase, urea, and high-sensitivity C reactive protein.*

# *Covariates in IPTW model: level of oxygen therapy, clinical classification, high-sensitivity C reactive protein and D-dimer levels, Platelet count at admission, CURB-65 score at hospital admission, and the highest level of oxygen therapy during hospitalization.*

*AC, anticoagulation; HR, hazard ratio; CI, confidence interval; IPTW, inverse probability of treatment weighting analysis; LMWH, low molecular weight heparin*.

Secondary outcomes included bleeding events and thrombocytopenia. By adjusting baseline covariates and time-dependent AC exposure, it was revealed that AC treatment was associated with higher risk of total bleeding events (adjusted HR = 3.187, 95% CI, 1.846 to 5.504, *P* < 0.001), CRNMB (adjusted HR = 3.713 95% CI, 1.446 to 9.532, *P* = 0.006) and microscopic hematuria (adjusted HR = 2.624, 95% CI, 1.280 to 5.380, *P* = 0.008), but not associated with major bleeding (*P* = 1.000) and thrombocytopenia (adjusted HR = 2.167, 95% CI, 0.750 to 6.263, *P* = 0.153) (Table 2). Considering death as a competing event, a competing risk model analysis using the Fine-Gray test was conducted to explore the association between AC and bleeding events or thrombocytopenia. Finally, the AC group had no significantly higher risk of bleeding events (Fine-Gray test, *P* = 0.500) or thrombocytopenia (Fine-Gray test, *P* = 0.911) than the non-AC group when using death as a competing event in the model.

### Clinical Subgroup Stratification

**Figure 3:**
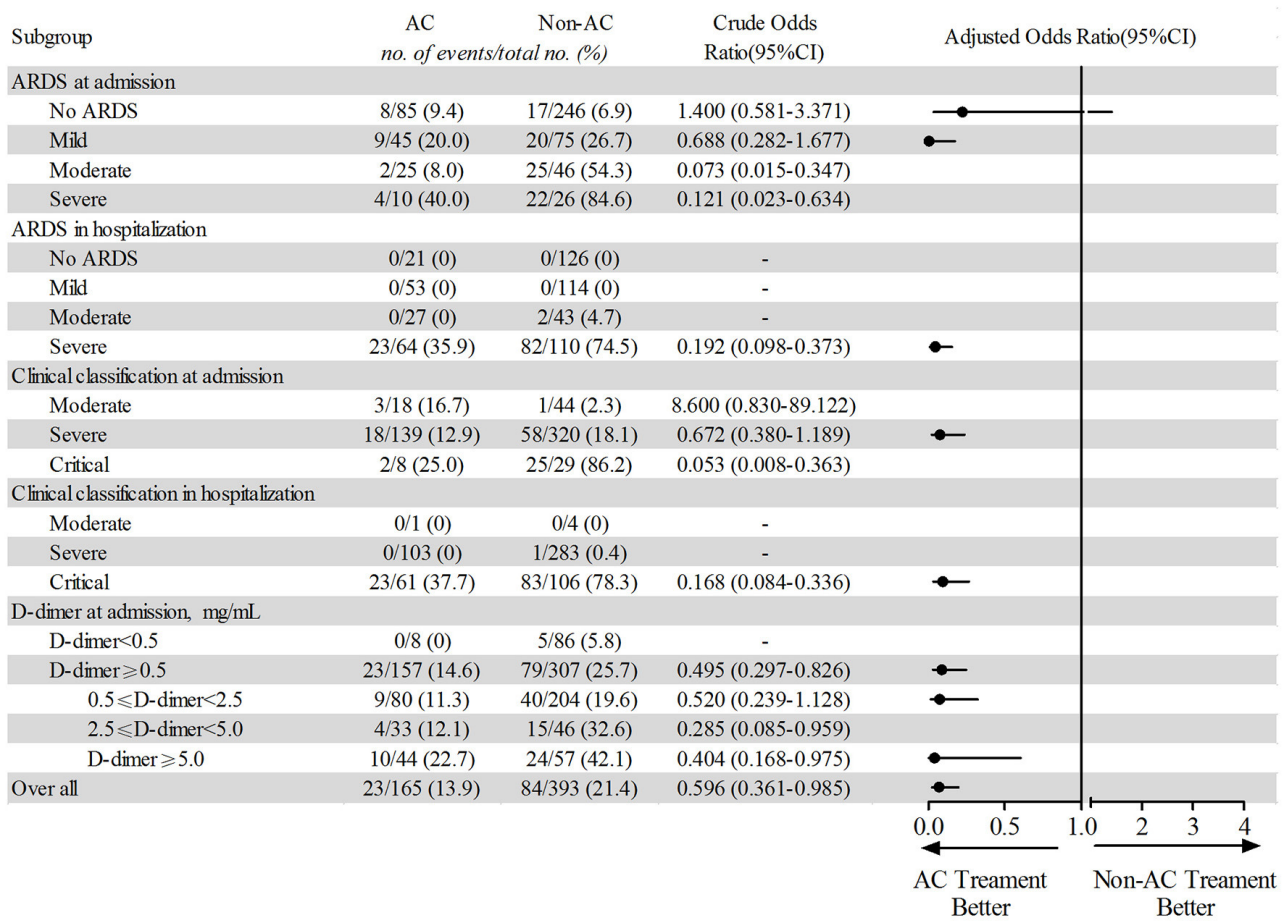
Subgroup analysis based on clinical stratification of in-hospital mortality between AC treatment and non-AC treatment patients. The multivariate logistic regression analysis adjusted for baseline covariates including age, smoking, comorbidities, white blood cells, D-dimer, lactate dehydrogenase, urea, antiviral therapy, intravenous immunoglobulin, convalescent plasma therapy, and oxygen therapy in hospitalization. ARDS, acute respiratory distress syndrome; AC, anticoagulation.

In-hospital mortality between the AC and the non-AC groups was compared in individuals stratified according to ARDS classification, COVID-19 clinical classification, and D-dimer levels at both hospital admission and during hospitalization. At hospital admission, AC treatment was associated with lower in-hospital mortality in subgroups of mild ARDS (adjusted odds ratio [OR] = 0.005, 95% CI, 0.000–0.174, *P* = 0.004), severe COVID-19 cases (adjusted OR = 0.076, 95% CI, 0.024–0.236, *P* < 0.001) and patients with a D-dimer level ≥0.5 μg/mL (adjusted OR = 0.042, 95% CI, 0.003–0.603, *P* = 0.020). During the hospital stay, AC treatment was associated with lower in-hospital mortality among patients who developed to severe ARDS (adjusted OR = 0.046, 95% CI, 0.013–0.157, *P* < 0.001) or critical COVID-19 (adjusted OR = 0.095, 95% CI, 0.034–0.266, *P* < 0.001) (Figure 3).

The interaction effect between AC treatment and the subgroups was also analyzed by logistic regression (Supplementary Table 2). An interaction effect was observed between the subgroups identified by ARDS at admission (*P* < 0.001) or clinical classification at admission (*P* < 0.001) and AC treatment.

### PAM Clustering Analysis

#### The Optimal Number of Clustering Determination

**Figure 4:**
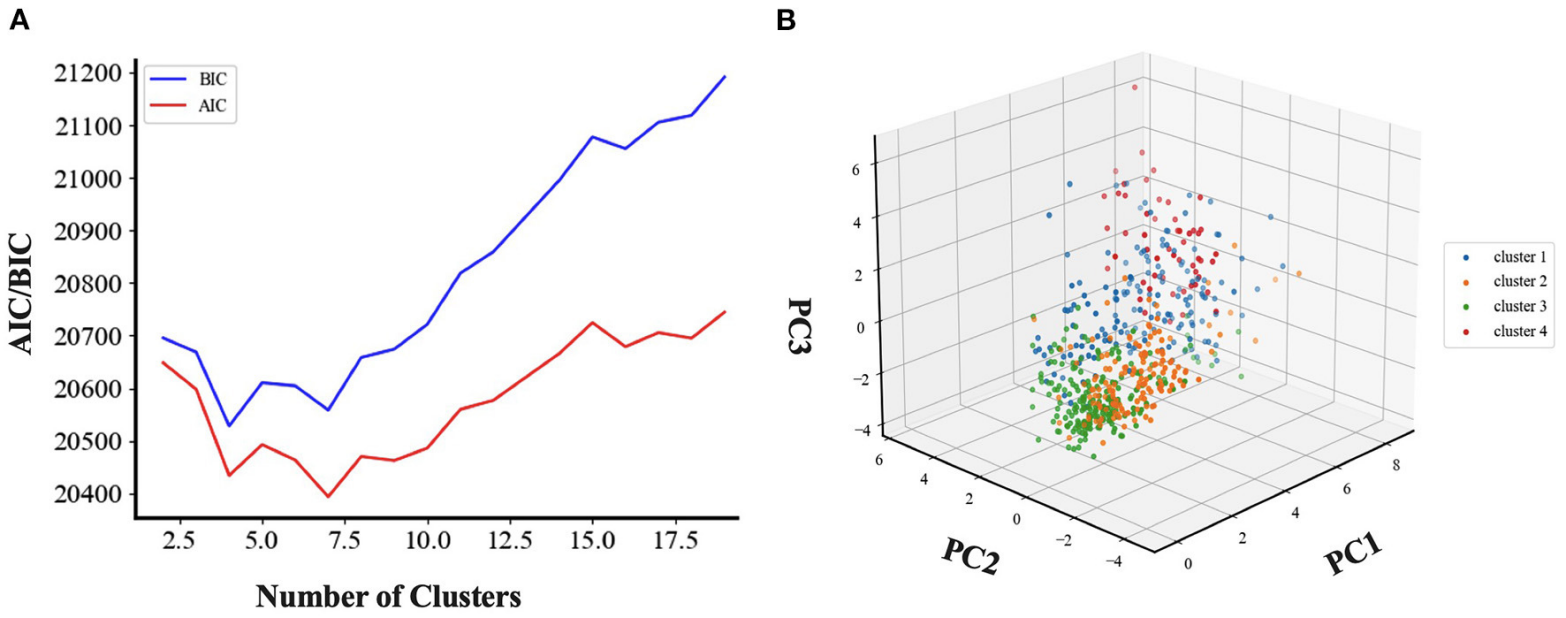
Model selection and cluster visualization. The selection of the best number of clusters K is based on BIC and AIC score. K = 4 was chosen after comparing the BIC score of models with different number of clusters by unsupervised clustering analysis. For AIC score, K = 4 is also a good choice for the trade-off between model complexity and the fitting of the data. **(A)** Unsupervised clustering analysis for choosing the best number of clusters. BIC, Bayesian information criteria; AIC, Akaike information criterion. **(B)** Three-dimensional visualization of clustering results. We visualize our clustering results in three-dimensional space. The high dimensional training data was projected into three-dimensional space by using principal component analysis. PC, Principal component.

BIC and AIC scores were used to automatically select the best number of clusters K for our model. As shown in Figure 4, we tried to find the optimal K by conducting an exhaustive search of the possible K values. By and large, the results from AIC and BIC are proportional. BIC score suggests that four clusters are optimal. For the AIC score, a shape “elbow point” is also indicated at four, after four the decrease is becoming notably smaller. Even though a better fitness of the data might be achieved by increasing the number of clusters, an additional cost will be needed including the over-fitting issue and the complexity of interpreting clinically plausible subgroups. Therefore, four clusters were selected to be optimal in this work.

#### Clinical Features of Patients' Subgroups

**Figure 5:**
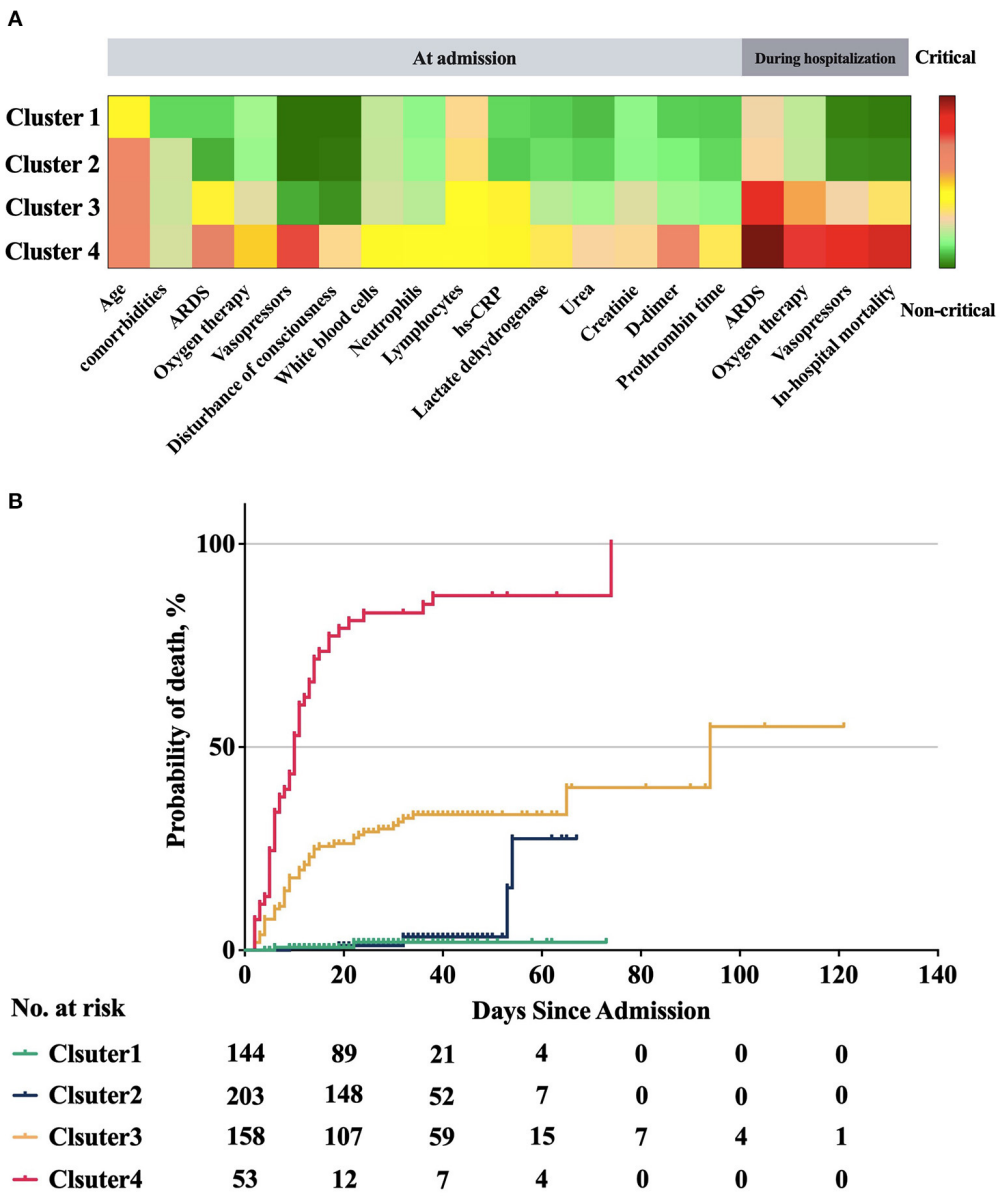
Clinical characteristics and probability of death of the four clusters. Clusters 1 and 2 were non-critical cases from admission to discharge and they showed lower probability of death. Cluster 3 had significant higher in hospital mortality and probability of death than clusters 1 and 2, but lower than that in cluster 4. Cluster 4 had the highest in hospital mortality and probability of death, and there were significant differences with the other three clusters. **(A)** Clinical characteristics of four clusters. Some of the significant clinical features are plotted for each cluster. The features are displayed by color-coded heatmap with normalized values. **(B)** Comparison of probability of death between four clusters. There was statistically significant difference in the survival distribution between any two groups (*P* < 0.001), except that there was no statistically significant difference in the survival distribution of cluster 1 and cluster 2 (Log-Rank test, *P* = 0.512 > 0.008).

The clinical features of the four clusters are shown in Tables 4, 5 and Figure 5. Patients in clusters 1 and 2 were non-critical cases. Patients in clusters 1 and 2 had no ARDS at admission then developed mild ARDS during the hospital stay. They mainly needed nasal cannula oxygenation at admission as well as during hospitalization. Within these non-critical clusters, cluster 1 was characterized by the youngest age (51 years, IQR 39–63) and had the least number of comorbidities (0, IQR 0–1). Compared to clusters 1 and 2, clusters 3 and 4 were critical cases. In laboratory testing results at admission, both clusters 3 and 4 had significantly higher neutrophils count, lower lymphocytes and platelets count, higher lactate dehydrogenase and hs-CRP, higher urea and creatinine, elevated prothrombin time and D-dimer. In vital signs, clusters 3 and 4 had significantly lower peripheral blood oxygen saturation and respiratory rate at admission. Either at admission or during the hospital stay, both clusters 3 and 4 had significantly more severe levels of ARDS and need a higher level of oxygen therapy. Notably, cluster 4 exhibited as the most critical sub-phenotype. Compared to cluster 3, patients in cluster 4 had significantly excessive inflammation (elevated white blood cells, neutrophils and lactate dehydrogenase), organ dysfunction (higher total bilirubin and urea), severe coagulopathy (elevated prothrombin time and D-dimer), unstable hemodynamics (higher rate of vasopressor use) and neurologic dysfunction (disturbance of consciousness). Compared to the other three clusters, cluster 4 had severe ARDS either at admission or during the hospital stay. As a result, cluster 4 also needed the highest level of oxygen therapy accordingly.

**Table 4:** Variables included in the PAM-based clustering model.

	**Cluster 1**	**Cluster 2**	**Cluster 3**	**Cluster 4**	***P*** **-value**
	**(*n* = 144)**	**(*n* = 203)**	**(*n* = 158)**	**(*n* = 53)**	
Age	51 (39, 63) abc	69 (62, 76)	67 (59, 74)	68 (63, 72)	<0.001
Gender	a	de			
Female	42 (29.2, −4.4)	156 (76.8, 11.4)	38 (24.1, −6.2)	15 (23.8, −2.6)	<0.001
Male	102 (70.8, 4.4)	47 (23.2, −11.4)	120 (75.9, 6.2)	38 (71.7, 2.6)	
No. of comorbidities	0 (0,1) abc	1 (0, 2)	1 (0, 2)	1 (1, 2)	<0.001
**Initial laboratory parameters**					
White blood cells, ×109/L	5.62 (4.47, 7.81) c	5.97 (4.89, 7.60) e	6.61 (4.72, 8.84) f	11.38 (8.96, 15.77)	<0.001
Neutrophils, ×109/L	3.79 (2.62, 5.44) bc	4.22 (3.03, 5.71) de	5.46 (3.47, 7.74) f	10.55 (7.64, 14.68)	<0.001
Lymphocytes, ×109/L	1.27 (0.90, 1.58) bc	1.06 (0.77, 1.42) de	0.61 (0.46, 0.82)	0.51 (0.36, 0.76)	<0.001
Platelets, ×109/L	283 (223, 342) abc	226 (177, 296) de	161 (127, 222)	157 (102, 216)	<0.001
Total bilirubin, mmol/L	8.9 (6.4, 12.1) bc	8.3 (6.4, 12.1) de	11.6 (8.3, 14.4) f	14.6 (10.6, 23.4)	<0.001
Lactate dehydrogenase, U/L	258 (205, 320) bc	284 (224, 344) de	444 (333, 524) f	601 (458, 795)	<0.001
Urea, mmol/L	3.95 (3.10, 5.28) bc	4.60 (3.30, 5.70) de	6.35 (4.70, 9.20) f	9.80 (6.60, 13.85)	<0.001
Creatinine, μmol/L	70 (58, 82) bc	63 (54, 78) de	83 (66, 101)	91 (75, 121)	<0.001
hs-CRP, mg/L	17.0 (6.7, 55.6) bc	17.0 (5.9, 43.3) de	109.8 (69.9, 162.3)	112.3 (71.4, 186.3)	<0.001
Prothrombin time, s	13.7 (13.2, 14.2) bc	13.8 (13.2, 14.3) de	14.5 (13.8, 15.4) f	16.2 (15.1, 17.6)	<0.001
D-dimer, mg/mL	0.91 (0.39, 2.10) bc	1.17 (0.58, 2.59) de	1.58 (0.86, 3.01) f	21.00 (2.72, 21.00)	<0.001
Hemoglobin, g/L	131 (120, 141) a	119 (109, 128) de	131 (120, 141)	132 (117, 142)	<0.001
**At admission**					
Oxygen therapy	bc	de	f		
Without oxygen inhalation	32 (22.2, 1.6)	47 (23.2, 2.4)	20 (12.7, −2.0)	1 (1.9, −3.2)	<0.001
Nasal cannula	105 (72.9, 1.7)	147 (72.4, 2.0)	103 (65.2, −0.6)	20 (37.7, −4.8)	
Face mask with reservoir bag	3 (2.1, −3.1)	8 (3.9, −2.8)	23 (14.6, 3.4)	12 (22.6, 4.0)	
High-flow nasal cannula	1 (0.7, 0)	0 (0, −1.5)	1 (0.6, −0.1)	2 (3.8, 2.8)	
NIV	2 (1.4, −1.6)	0 (0, −3.4)	6 (3.8, 0.2)	12 (22.6, 7.8)	
IMV	1 (0.7, −1.5)	1 (0.5, −2.2)	5 (3.2, 0.8)	6 (11.3, 4.6)	
Vasopressor	0 (0, −1.9) c	0 (0, −2.4) e	2 (1.3, −0.6) f	8 (15.1, 7.7)	<0.001
Disturbance of consciousness	0 (0, −3.0) c	2 (1.0, −2.9) e	6 (3.8, −0.4) f	16 (30.2, 9.8)	<0.001
SpO_2_	97 (95, 98) bc	97 (95, 99) de	93 (86, 96)	90 (79, 96)	<0.001
Respiratory rate, /min	20 (20, 22) bc	20 (20, 22) de	20 (20, 25)	25 (20, 32)	<0.001
Heart rate, /min	92 (84, 105) a	86 (77, 99) d	96 (86, 109)	88 (78, 113)	<0.001
MAP, mmHg	97 (90, 104)	97 (89, 106)	96 (89, 107)	99 (91, 107)	0.771
**During hospitalization**					
Oxygen therapy	bc	de	f		
Without oxygen inhalation	4 (2.8, 2.3)	2 (1.0, −0.2)	0 (0, −1.5)	0 (0, −0.8)	<0.001
Nasal cannula	121 (84.0, 7.1)	173 (85.2, 9.6)	34 (21.5, −11.2)	0 (0, −9.1)	
Face mask with reservoir bag	8 (5.6, −1.1)	13 (6.4, −0.9)	22 (13.9, −0.9)	0 (0, −2.2)	
High-flow nasal cannula	4 (2.8, −1.1)	6 (3.0, −1.3)	14 (8.9, 3.1)	1 (1.9, −1.0)	
NIV	3 (2.1, −4.8)	1 (0.5, −7.0)	57 (36.1, 9.3)	18 (34.0, 4.3)	
IMV	3 (2.1, −4.5)	8 (3.9, −4.8)	27 (17.1, 1.9)	34 (64.2, 11.7)	
ECMO	1 (0., −0.3)	0 (0, −1.7)	4 (2.5, 2.6)	0 (0, −0.7)	
Vasopressor	3 (2.1, −5.7) bc	7 (3.4, −6.6) de	43 (27.2, 3.8) f	45 (84.9, 13.5)	<0.001
Lowest SpO_2_	95 (93, 96) bc	95 (92, 96) de	89 (77, 94) f	67 (56, 80)	<0.001

*Quantitative variables are expressed as medians (interquartile ranges). Categorical variables are expressed as No. (%, adjusted standardized residuals). It is considered that the difference between the actual frequency and the expected frequency of this value is statistically significant if the absolute value of the adjusted standardized residual is >2. Bonferroni correction for multiple comparison was used in the comparison of cluster analysis results. The letters “a” to “f” indicate a significant difference between two group, respectively (a = cluster 1 vs. cluster 2, b = cluster 1 vs. cluster 3, c = cluster 1 vs. cluster 4, d = cluster 2 vs. cluster 3, e = cluster 2 vs. cluster 4, f = cluster 3 vs. cluster 4. PAM, partitioning around medoids; hs-CRP, high sensitivity C-reactive protein; NIV, noninvasive ventilation (bi-level); IMV, invasive mechanical ventilation; SpO_2_, peripheral blood oxygen saturation; MAP, mean arterial pressure; ECMO, extracorporeal membrane oxygenation*.

**Table 5:** Outcome and variables not included in the PAM-based clustering model.

	**Cluster 1**	**Cluster 2**	**Cluster 3**	**Cluster 4**	***P*** **-value**
	**(*n* = 144)**	**(*n* = 203)**	**(*n* = 158)**	**(*n* = 53)**	
**Symptoms**					
Fever	120 (83.3, 0.4)	159 (78.3, −1.8)	135 (85.4, 1.2)	45 (84.9, 0.5)	0.306
Cough	109 (75.7, 0.1)	145 (71.4, −1.6)	123 (77.8, 0.9)	43 (81.1, 1.0)	0.367
Expectoration	66 (45.8, 0.3)	86 (42.4, −0.8)	75 (47.5, 0.8)	22 (41.5, −0.5)	0.746
Shortness of breath	74 (51.4, −1.7)	117 (57.6, 0.1)	100 (63.3, 1.8)	29 (54.7, −0.4)	0.210
Myalgia	27 (18.8, −0.8)	38 (18.7, −1.0)	41 (25.9, 1.8)	11 (20.8, 0)	0.332
Fatigue	48 (33.3, −0.3)	68 (33.5, −0.3)	57 (36.1, 0.5)	19 (35.8, 0.2)	0.942
Diarrhea	23 (16.0, −2.2)	50 (24.6, 0.9)	39 (24.7, 0.7)	14 (26.4, 0.7)	0.177
Nausea/vomiting	17 (11.8, −0.7)	29 (14.3, 0.4)	23 (14.6, 0.5)	6 (11.3, −0.5)	0.842
**At admission**					
FiO_2_	0.30 (0.27, 0.30) bc	0.30 (0.27, 0.30) de	0.36 (0.30, 0.42) f	0.51 (0.38, 0.63)	<0.001
SBP, mmHg	124 (117, 136) ac	131 (121, 143)	131 (120, 146)	135 (122, 151)	0.004
DBP, mmHg	81 (76, 90)	79 (72, 87)	78 (73, 88)	80 (72, 88)	0.258
CURB-65 score	0 (0, 1) abc	1 (1, 1) de	1 (1, 2) f	2 (2, 3)	<0.001
ARDS at admission	bc	de	f		
No ARDS	108 (75.0, 4.4)	170 (83.7, 8.9)	49 (31.0, −8.6)	4 (7.5, −8.1)	<0.001
Mild	29 (20.1, −0.5)	26 (12.8, −3.8)	55 (34.8, 4.8)	10 (18.9, −0.5)	
Moderate	5 (3.5, −3.9)	6 (3.0, −5.2)	41 (25.9, 5.9)	19 (35.8, 5.3)	
Severe	2 (1.4, −2.9)	1 (0.5, −4.3)	13 (8.2, 1.1)	20 (37.7, 9.7)	
**Initial laboratory parameters**					
Hematocrit, %	38 (34, 40) a	35 (32, 38) de	37 (34, 40)	38 (34, 41)	<0.001
ALT, U/L	28 (19, 48) a	20 (13, 36) d	29 (17, 47)	24 (19, 41)	<0.001
AST, U/L	27 (20, 38) bc	23 (18, 34) de	40 (28, 62)	41 (26, 56)	<0.001
Total protein, g/L	69.6 (66.2, 73.5) bc	68.0 (64.6, 72.7) de	65.9 (62.5, 70.5)	63.3 (60.8, 69.9)	<0.001
Albumin, g/L	35.7 (31.5, 39.5) bc	34.4 (31.4, 38.3) de	31.4 (28.4, 34.2)	29.3 (26.7, 31.8)	<0.001
Globulins, g/L	33.3 (29.9, 37.8) b	33.5 (30.1, 37.1) d	34.9 (31.4, 39.1)	34.8 (31.5, 39.1)	0.003
**During hospitalization**					
Highest FiO_2_	0.30 (0.30, 0.36) bc	0.33 (0.30, 0.36) de	0.66 (0.45, 1.00) f	1.00 (0.70, 1.00)	<0.001
ARDS during hospitalization	bc	de	f		
No ARDS	62 (43.1, 5.3)	83 (40.9, 5.9)	2 (1.3, −8.5)	0 (0, −4.6)	<0.001
Mild	60 (41.7, 3.6)	80 (39.4, 3.7)	27 (17.1, −4.2)	0 (0, −5.0)	
Moderate	11 (7.6, −2.1)	22 (10.8, −0.9)	35 (22.2, 4.3)	2 (3.8, −2.0)	
Severe	11 (7.6, −7.1)	18 (8.9, −8.6)	94 (59.5, 9.1)	51 (96.2, 10.7)	
In-hospital mortality	2 (1.4, −6.3) bc	6 (3.0, −7.4) de	52 (32.9, 5.2) f	47 (88.7, 13.5)	<0.001
Survival time, days	23 (17, 34) abc	31 (18, 40) e	31 (14, 44) f	10 (6, 17)	<0.001
Bleeding events	21 (14.6, −2.4) bc	31 (15.3, −2.8) de	51 (32.3, 3.8)	18 (34.0, 2.3)	<0.001
Major bleeding	0 (0, −1.2)	0 (0, −1.5)	4 (2.5, 3.2)	0 (0, −0.7)	0.021
CRNMB	6 (4.2, −0.9)	7 (3.4, −1.8)	14 (8.9, 2.0)	5 (9.4, 1.2)	0.070
Bloody sputum	2 (1.4, −0.2)	2 (1.0, −0.9)	5 (3.2, 1.8)	0 (0, −1.0)	0.378
Microscopic hematuria	12 (8.3, −2.4) bc	21 (10.3, −2.0) de	34 (21.5, 3.0)	13 (24.5, 2.2)	<0.001
Positive FOBT/GOBT	1 (0.7, −0.3)	2 (1.0, 0.2)	1 (0.6, −0.4)	1 (1.9, 0.8)	0.713
Thrombocytopenia	5 (3.5, −4.3) bc	7 (3.4, −5.5) de	37 (23.4, 3.9) f	30 (56.6, 9.3)	<0.001

*Quantitative variables are expressed as medians (interquartile ranges). Categorical variables are expressed as No. (%, adjusted standardized residuals). It is considered that the difference between the actual frequency and the expected frequency of this value is statistically significant if the absolute value of the adjusted standardized residual is >2. Bonferroni correction for multiple comparison was used in the comparison of cluster analysis results. The letters “a” to “f” indicate a significant difference between two group respectively (a = cluster 1 vs. cluster 2, b = cluster 1 vs. cluster 3, c = cluster 1 vs. cluster 4, d = cluster 2 vs. cluster 3, e = cluster 2 vs. cluster 4, f = cluster 3 vs. cluster 4. PAM, partitioning around medoids; FiO_2_, fraction of inspired oxygen; SBP, Systolic blood pressure; DBP, Diastolic blood pressure; ARDS, acute respiratory distress syndrome; ALT, Alanine aminotransferase; AST, Aspartate aminotransferase; CRNMB, clinically relevant non-major bleeding; FOBT, fecal occult blood test; GOBT, gastric occult blood test*.

#### AC Treatment and Outcomes in Different Patients Clusters

**Figure 6:**
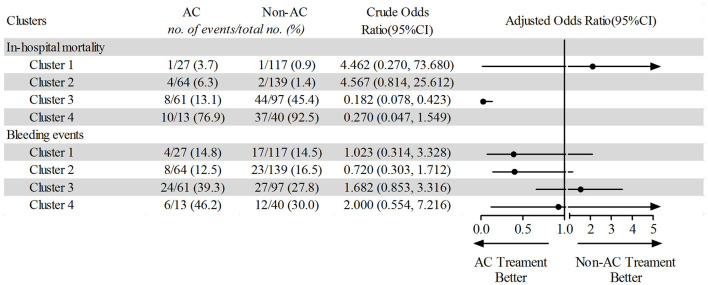
Comparing of in-hospital mortality and bleeding events between AC treatment and non-AC treatment patients based on unsupervised machine learning. AC, anticoagulation.

In the non-critical cluster (clusters 1 and 2), we found no significantly lower in-hospital death risk associated with AC treatment (Figure 6). In cluster 3, patients who received AC treatment had a significantly lower in-hospital mortality than those who did not receive this treatment (adjusted OR = 0.027, 95% CI, 0.005 to 0.134, *P* < 0.001). However, patients in cluster 4, who had elevated inflammation biomarkers and even severe multi-organ dysfunction, did not benefit from AC treatment. For safety endpoint, AC treatment was not associated with increasing bleeding events (Figure 6). In addition, the interaction effect was found between the AC treatment and sub-phenotypes identified by PAM clustering (*P* < 0.001, Supplementary Table 2).

## Discussion

COVID-19 infections have affected patients globally. The ISTH pointed out that COVID-19 patients develop a clinically significant coagulopathy, characterized by thrombocytopenia, mildly prolonged prothrombin time, and elevated serum D-dimer levels (27). Recent research indicates that coagulopathy is not only common in COVID-19 patients but is also associated with increased mortality (9). The potential mechanism for the development of coagulopathy in COVID-19 patients may be related to endothelial cell dysfunction (37) and hypoxia-induced thrombosis (38) following a SARS-CoV-2 infection. Because the endothelium plays an important role in regulating hemostasis, fibrinolysis, and vessel wall permeability, endothelial dysfunction in pulmonary microvessels may act as a trigger for immunothrombosis, resulting in coagulopathy. Histological analysis of pulmonary vessels in COVID-19 patients shows more widespread thrombosis with microangiopathy compared to that observed in patients with influenza. Based on this preliminary evidence, AC treatment may be beneficial for COVID-19 patients by inhibiting thrombin generation and thereby reducing mortality. The ISTH suggests that a prophylactic dose of LMWH should be considered in all patients without contraindications. Moreover, the Chinese Diagnosis and Treatment Protocol for COVID-19 Patients (Version 8.0) also suggested using AC treatment in selected patients. However, these recommendations require additional clinical evidence to determine the association between AC treatment and the outcome of COVID-19 patients, and also to clarify the indications, contradictions and optimal duration, dose, and time to use AC. We conducted this matched cohort study using a comprehensive source of COVID-19 patients. In general, results showed that receiving AC treatment was associated with a decreased in-hospital mortality in these patients. Although patients who received AC treatment exhibited a significant increase in CRNMB and microscopic hematuria, they had no increase in the incidence of major bleeding. A clinical subgroup analysis was also carried out to identify patient subgroups who receive greater benefit from AC treatment. At hospital admission, patients of severe COVID-19 clinical cases, patients with mild ARDS or patients who had a D-dimer level ≥0.5 μg/mL were more likely to benefit from AC therapy. During hospitalization, patients who developed severe ARDS or critical COVID-19 cases were more likely to benefit from AC therapy (Figure 3). Results of clusters identified by unsupervised machine learning revealed similar results as of clinical subgroups. Critical patients of cluster 3 could benefit from AC treatment whereas non-critical patients in clusters 1 and 2 did not. Nevertheless, a sub-phenotype (cluster 4) exhibited even severe multiple organ dysfunction and excessive inflammation might not benefit from AC therapy.

To date, several research works have investigated systemic AC therapy in COVID-19 patients (9–13, 39). Although the results generally suggested that AC treatment was associated with lower mortality of COVID-19 patients, a constant instruction for clinic application was not easy to conclude. Several possible reasons are worth to be noted. As a retrospective cohort study, imbalance of baseline covariates, covariates related to outcome and covariates related to exposure assignment might lead to biased results. Among the existing studies, some studies used propensity score methods for reducing the effects of confounding (12, 39), some did not (9, 10). In our study, we applied PSM which yield a relatively balanced cohort. We also did IPTW analysis, another propensity score method, to detect the selective bias potentially caused by PSM in the full cohort. The results from PS matched cohort and IPTW analysis in the full cohort both revealed that AC treatment was associated with lower death risk. We also noted that, without the PS method, either the crude results or baseline-adjusted results will lead to an adverse conclusion. Immortal time is a gap period between exposure (usually the span after cohort follow-up) and initiation of follow-up (20). This might cause potential immortal time bias and exaggerate the association between the exposure and outcome. As a result, we carried out a Cox proportional hazards model with a time-dependent manner for the drug exposure in this study.

Another key question concerns the confounders involving various durations, dosages and types of AC treatment. In a retrospective cohort study from the Mount Sinai Health System (11), the duration of hospitalization (median 5 days, IQR 3–8 days) and the course of AC treatment (median 3 days, IQR 2–7 days) were relatively short. Within the current consensus on anticoagulant therapy for venous thromboembolism, it is generally considered that patients with confirmed deep vein thrombosis or pulmonary embolism need LMWH treatment for at least 5 days followed by Dabigatran or Edoxaban (40). As a new disease without comprehensive study until now, to determine the optimal duration, type and dosage of AC treatment need more evidence. In our study, we conducted a series of sensitivity analyses to investigate the relationship between outcome and AC treatment duration, dosage and type. We found that AC treatment for 7 days or longer was associated with a lower death risk while AC treatment for <7 days was not. Low dose thromboprophylaxis, intermediate dose thromboprophylaxis and therapeutic dose anticoagulation were all associated with lower death risk. Although we recorded detailed AC treatment type, the majority was LMWH (Supplementary Figure 1), we only investigated LMWH and non-LMWH for sensitivity analyses here. It is revealed that both LMWH and non-LMWH treatment were associated with a lower death rate.

The heterogeneity of the research population is another vital confounder that influences the results. In our study, we analyzed a full cohort of unselected patients from two designated hospitals including mild to critical cases. In general, we found that AC treatment was associated with low mortality, which had a constant result with the previous studies. Furtherly, we investigated who might benefit from AC treatment in subgroup analyses. The stratification criteria used in our study included the most frequently used classification of clinical severity of the COVID-19 patients. It is well-known that hypoxia is a core clinical manifestation and major pathophysiology change that contributes to the death of COVID-19 patients (41–43). The classification of both ARDS (18, 29, 44) and COVID-19 clinical severity classification (17, 45) indicates the severity of hypoxia and accordingly, they are used frequently by clinicians to evaluate and triage patients and to decide major treatments (e.g., levels of oxygen therapy). As a result, we stratified patients according to ARDS classification, COVID-19 clinical classification, and D-dimer levels at both hospital admission and during hospitalization. By this strategy, we found that, at admission, severe cases of COVID-19 clinical classification, mild ARDS cases and patients with a D-dimer level ≥0.5 μg/mL may benefit from AC. While during the hospital stay, critical cases and severe ARDS cases may benefit from AC. These results were in constant with Sun et al.' study (9) with severe cases and subgroup analysis from Shen et al. (39) and CORIST Studies (12).

Clustering the study population may help minimize the influence of heterogeneity on the results. The traditional way to categorize patients is based on pre-defined standards. The standards are usually defined by a group of experienced experts with a strong background and prior knowledge in the medical area. Therefore, the procedure for generating the standards alone takes considerable effort and time. In addition, these standards cannot easily be quickly established or updated for a new situation in a short period, which was apparent when we faced this new pandemic, COVID-19. Unsupervised clustering algorithms in machine learning offer another perspective to perform the identification of data subclasses. Unsupervised clustering approaches can achieve more stable and robust clustering results without any prior knowledge of the meaning of each variable in the data. In addition, it may also identify some intrinsic correlations between the variables which sometimes cannot be easily noticed by human experts. Considering the heterogeneity of COVID-19 patients, an innovative strategy was carried out to identify subphenotype of patients who exhibited distinct clinical characteristics and respond to certain treatment using unsupervised learning approach (46). To this end, a four-class PAM-based clustering model was established, representing four distinct COVID-19 patient subphenotypes with different clinical characteristics. In particular, clusters 1 and 2 were non-critical cases with significantly lower mortality. Patients in these two clusters did not benefit from AC treatment. Clusters 3 and 4 were critical cases both exhibited significant abnormal laboratory testing results at admission and unstable vital sign. Cluster 3 had mild to moderate ARDS at admission and progressed to severe ARDS during the hospital stay. Patients in cluster 3 can benefit from AC treatment and had no significant increase in bleeding events. Compared to cluster 3, cluster 4 was the most critical cases and has the highest mortality. A novel result by the clustering approach was that, among these most critical patients (clusters 4), who had moderate or severe ARDS at admission and developed severe ARDS during the hospital stay, AC treatment was not associated with a lower death risk. Further characteristic analysis of these clusters revealed cluster 4 was characterized by multiple organ dysfunction and excessive inflammation. This led us to conclude that critical COVID-19 patients with these features cannot benefit from AC treatment. Recently, an open-label, adaptive, multiplatform, randomized control trial was published (14), with the researchers noting that the initial strategy of therapeutic-dose anticoagulation did not result in a greater probability of survival in critically ill COVID-19 patients (defined as COVID-19 that led to the receipt of ICU-level respiratory or cardiovascular organ support in an ICU) compared to usual-care thromboprophylaxis. This result was different from our clinical subgroup analysis but similar to the phenotypes of clusters 4 in our unsupervised clustering analysis.

Analysis of safety endpoints showed that although the risk of bleeding events, including CRNMB and microscopic hematuria, were higher in the AC group compared to the non-AC group, there was no significant difference in the risk of major bleeding events or thrombocytopenia between the two groups. In brief, the above findings suggested that the use of AC treatment for 7 days or longer in hospitalized COVID-19 patients was associated with increased CRNMB and microscopic hematuria but not with other bleeding events, especially major bleeding. These key observations are consistent with those reported in recent studies (11, 39). Although the competing risk model analysis in this study revealed that there was no significant difference in bleeding risk between the AC and non-AC groups when considering death as a competitive event, the increase of CRNMB still reminds clinicians should be more cautious when using anticoagulation treatment.

This study had several limitations. Firstly, as a retrospective cohort study, imbalanced confounders and selective bias may exist. Large-scale, multicenter, randomized, controlled trials are urgently needed to fully assess the efficacy of AC in patients with COVID-19. Besides, our unsupervised clustering model did not take the importance of each variable into consideration, as it treated all the variables equally as numerical values and measured the similarity between patients based on geometric distance. However, the variables could have completely different semantic meanings. Therefore, it is still necessary for human experts to inspect the clustering results to make sure that the results are explainable. Future work could be to intrinsically integrate the importance of clinic variables into the similarity measurements of unsupervised cluster models.

## Conclusion

COVID-19 patients who received AC treatment for 7 days or longer had a significantly lower in-hospital death risk but not higher risk of major bleeding. Through the clinical subgroup analysis, critically ill patients were more likely to benefit from AC treatment. Specifically, the unsupervised machine learning model revealed that, within critically ill COVID-19 patients, patients characterized by multiple organ dysfunction (neurologic, circulation, coagulation, kidney and liver dysfunction) and excessive inflammation may not benefit from AC treatment.

## Data Availability Statement

The raw data supporting the conclusions of this article will be made available by the authors, without undue reservation.

## Ethics Statement

This retrospective cohort study was approved by the ethics committee of Tongji Medical College, Huazhong University of Science and Technology (No. 2020-S220). The clinical trial was registered and verified by the Chinese Clinical Trial Registry (ChiCTR2000039855).

## Author Contributions

YB, YL, SW, and SL conceptualized the paper. YB, ZH, and SL had full access to all of the data in the study and take responsibility for the integrity of the data and the accuracy of the data analysis. HD and JC performed the unsupervised clustering analysis by machine learning and established the clustering model. YB, YL, ZH, YFa, GY, SY, YW, JL, YFe, LX, YZ, and ZY contributed to the acquisition, analysis, and interpretation of data. YB and YL drafted the manuscript. HD and JC drafted the clustering model part of the manuscript. PZ and SL helped revise the manuscript. YB, SW, and JH did the statistical analysis. SL and YB obtained the fundings. SL attests that all listed authors meet authorship criteria and that no others meeting the criteria have been omitted. YB and SL supervised the study and are the guarantors. All authors contributed to the critical revision of the manuscript for important intellectual content and gave final approval of the version to be published.

## Funding

This study was supported by the COVID-19 Rapid Response Research Project of Huazhong University of Science and Technology (Grant 2020kfyXGYJ049, to SL), and the Natural Science Foundation of Hubei Province (Grant No. 2021CFB376, to YB).

## Conflict of Interest

The authors declare that the research was conducted in the absence of any commercial or financial relationships that could be construed as a potential conflict of interest.

## Publisher's Note

All claims expressed in this article are solely those of the authors and do not necessarily represent those of their affiliated organizations, or those of the publisher, the editors and the reviewers. Any product that may be evaluated in this article, or claim that may be made by its manufacturer, is not guaranteed or endorsed by the publisher.
