# Supplementary material for: Efficacy and Safety of Anticoagulation Treatment in COVID-19 Patient Subgroups Identified by Clinical-Based Stratification and Unsupervised Machine Learning: A Matched Cohort Study
Source: Front Med (Lausanne). 2021 Dec 24;8:786414. doi: 10.3389/fmed.2021.786414 (PMC8740912; doi:10.3389/fmed.2021.786414)
Supplement: Supplementary file 1 [file Data_Sheet_1.docx]

**Figure S1. Details of** **anticoagulation regimens**

**
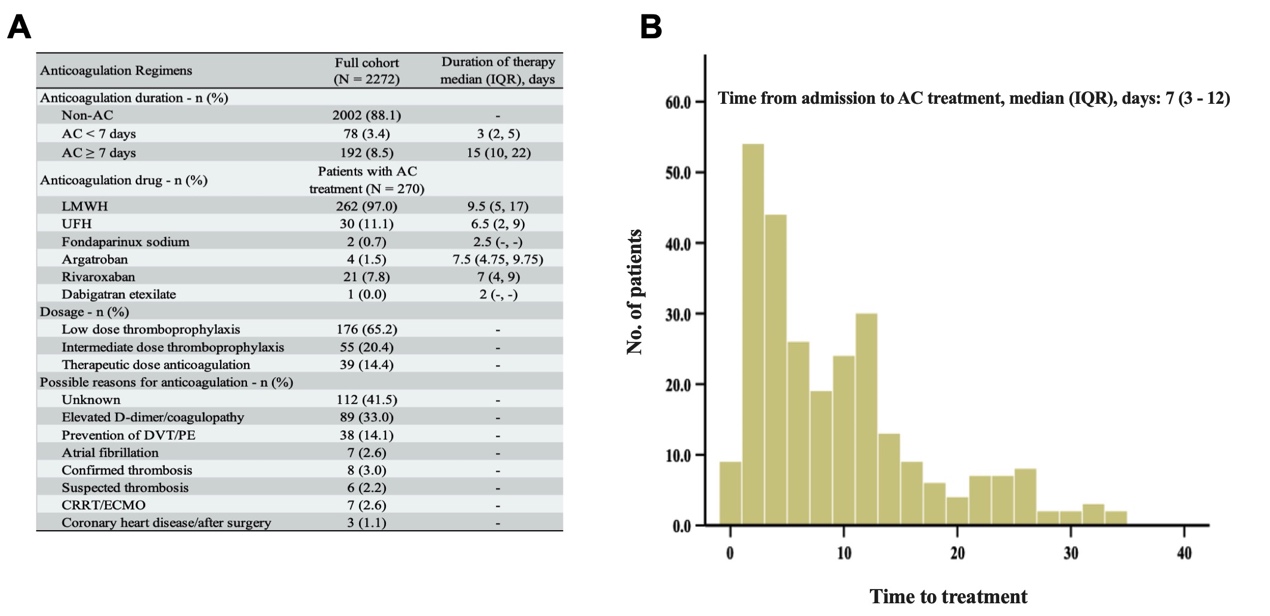
**

(A) Anticoagulation regimens. Detailed AC treatment type, dosage, duration, time of initiation from admission and possible reasons for AC treatment were collected among patients who received AC during hospitalization (n = 270). A part of patients received more than two anticoagulation drugs. The majority of anticoagulation drugs is LMWH. Doses of AC used in this study were categorized according to the AC doses defined in the randomized clinical trial of REMAP-CAP, ACTIV-4a, and ATTACC [1]. All decisions were made by the bedside physicians, which included whether and why to use anticoagulation.

(B) Histogram of time (in days) from admission to first dose of anticoagulation drug for those patients that received AC treatment. Study baseline is defined as hospital admission.

Abbreviation: AC, anticoagulation

| Table S1. Definition of ARDS and COVID-19 clinical classification | | |
| --- | --- | --- |
| ARDS classification by Berlin Definition | | |
|  | Mild | 200 mm Hg < PaO_2_ / FiO_2_ ≤ 300 mm Hg with PEEP or CPAP ≥ 5 cm H_2_O ^a^ |
|  | Moderate | 100 mm Hg < PaO_2_ / FiO_2_ ≤ 200 mm Hg with PEEP ≥ 5 cm H_2_O |
|  | Severe | PaO_2_ / FiO_2_ ≤ 100 mm Hg with PEEP ≥ 5 cm H_2_O |
| ARDS classification by Kigali modification of the Berlin criteria | | |
|  | Mild | SpO_2_ / FiO_2_ ≤ 315 and >235 |
|  | Moderate | SpO_2_ / FiO_2_ ≤ 235 and >150 |
|  | Severe | SpO_2_ / FiO_2_ ≤ 150 |
| COVID-19 clinical classification | | |
|  | Mild | The clinical symptoms are mild, and there is no pneumonia manifestation in imaging tests. |
|  | Moderate | Fever, respiratory symptoms, other clinical manifestations, and pneumonia can be seen on imaging. |
|  | Severe | Meeting any of the following:  (1) Shortness of breath, RR ≥ 30 times/min; (2) In the resting state, during inhalation, the oxygen saturation is ≤93%; (3) Arterial partial pressure of oxygen (PaO_2_)/inhaled oxygen concentration (FiO_2_) ≤ 300mmHg (1mmHg = 0.133kPa) ^a^;  (4) The clinical symptoms are progressively worsening, and lung imaging shows that within 24 to 48 hours the lesion has progressed significantly > 50%. |
|  | Critical | Meeting one of the following conditions: (1) Respiratory failure and mechanical ventilation are required; (2) Shock; (3) Other organ failures, ICU monitoring and treatment is required. |
| *^a^ In areas with high altitude (more than 1000 meters above sea level), PaO2/FiO2 should be adjusted according to the following formula: PaO2/FiO2 × [760/atmospheric pressure (mmHg)]. Abbreviation: ARDS, acute respiratory distress syndrome; PaO2, arterial partial pressure of oxygen; FiO_2_, fraction of inspired oxygen; PEEP, positive end-expiratory pressure; CPAP, continuous positive airway pressure; SpO_2_, peripheral oxygen saturation; RR, respiratory rate; ICU, intensive care unit.* | | |

| Table S2. Interaction between AC treatment and subgroups in logistic regression | | |
| --- | --- | --- |
| Variable | OR (95%CI) | *P*-value |
| AC treatment | 1.442 (0.672 - 3.093) | 0.347 |
| ARDS at admission | 4.086 (3.015 - 5.537) | <0.001 |
| (AC treatment) * (ARDS at admission) | 0.368 (0.215 - 0.628) | <0.001 |
| AC treatment | - | 0.995 |
| ARDS in hospitalization | 65.725 (15.981 - 270.307) | <0.001 |
| (AC treatment) * (ARDS in hospitalization) | - | 0.995 |
| AC treatment | 6316.786 （68.950 - 578704.654） | <0.001 |
| Clinical classification at admission | 23.511 （8.875 - 62.285） | <0.001 |
| (AC treatment) * (Clinical classification at admission) | 0.048 （0.011 - 0.216） | <0.001 |
| AC treatment | - | 0.997 |
| Clinical classification in hospitalization | 1017.681 (135.407 - 7648.584) | <0.001 |
| (AC treatment) * (Clinical classification in hospitalization) | - | 0.997 |
| AC treatment | 0.769 (0.147 - 4.031) | 0.756 |
| D-dimer at admission | 2.004 (1.551 - 2.590) | <0.001 |
| (AC treatment) * (D-dimer at admission) | 0.812 (0.470 - 1.403) | 0.456 |
| AC treatment | 0.066 (0.021 - 0.210) | <0.001 |
| PAM clustering | 0.055 (0.028 - 0.110) | <0.001 |
| (AC treatment) * (PAM clustering) | 5.497 (2.218 - 13.625) | <0.001 |
| *OR, odds ratio; CI, Confidence interval; AC, anticoagulation; ARDS, acute respiratory distress syndrome; PAM, partitioning around medoids* | | |

**Reference**

[1] Investigators R-C, Investigators AC-a, Investigators A, Goligher EC, Bradbury CA, McVerry BJ, et al. Therapeutic Anticoagulation with Heparin in Critically Ill Patients with Covid-19. N Engl J Med. (2021) 385(9):777-789. doi:10.1056/NEJMoa2103417.
